# Post-marketing surveillance data of thrombomodulin alfa: sub-analysis in patients with sepsis-induced disseminated intravascular coagulation

**DOI:** 10.1186/2052-0492-2-30

**Published:** 2014-04-30

**Authors:** Yutaka Eguchi, Satoshi Gando, Hiroyasu Ishikura, Daizoh Saitoh, Jun Mimuro, Hoyu Takahashi, Isao Kitajima, Hajime Tsuji, Tadashi Matsushita, Ryuichi Tsujita, Osamu Nagao, Yoichi Sakata

**Affiliations:** Department of Critical and Intensive Care Medicine, Shiga University of Medical Science, Shiga, Japan; Division of Acute and Critical Care Medicine, Department of Anesthesiology and Critical Care Medicine, Hokkaido University Graduate School of Medicine, Hokkaido, Japan; Department of Emergency and Critical Care Medicine, Faculty of Medicine, Fukuoka University, Fukuoka, Japan; Division of Traumatology, National Defense Medical College Research Institute, National Defense Medical College, Saitama, Japan; Division of Cell and Molecular Medicine, Center for Molecular Medicine, Jichi Medical University, School of Medicine, Tochigi, Japan; Department of Internal Medicine, Niigata Prefectural Kamo Hospital, Niigata, Japan; Department of Clinical Laboratory and Molecular Pathology, Graduate School of Medical and Pharmaceutical Science, University of Toyama, Toyama, Japan; Department of Blood Transfusion, Kyoto Prefectural University of Medicine, Kyoto, Japan; Department of Transfusion Medicine, Nagoya University Hospital, Aichi, Japan; ART Project, Pharmaceuticals Sales Division, Asahi Kasei Pharma Corporation, Tokyo, Japan; Post-Marketing Surveillance Dept. Reliability Assurance Center, Asahi Kasei Pharma Corporation, Tokyo, Japan; The Japanese Society on Thrombosis and Hemostasis Post-Marketing Surveillance Committee for Recomodulin® Injection, Tokyo, Japan

**Keywords:** Anticoagulant, JAAM criteria, Sepsis, SIRS, SOFA score

## Abstract

**Background:**

Thrombomodulin alfa (TM-α, recombinant thrombomodulin) significantly improved disseminated intravascular coagulation (DIC) when compared with heparin therapy in a phase III study. Post-marketing surveillance of TM-α was performed to evaluate the effects and safety in patients with sepsis-induced DIC.

**Methods:**

From May 2008 to April 2010, a total of 1,787 patients with sepsis-induced DIC treated with TM-α were registered. DIC was diagnosed based on the Japanese Association for Acute Medicine (JAAM) criteria. The DIC resolution and survival rates on day 28 after the last TM-α administration, and changes in DIC, systemic inflammatory response syndrome (SIRS), and sequential organ failure assessment (SOFA) scores and coagulation and inflammation markers were evaluated.

**Results:**

The most frequent underlying disease was infectious focus-unknown sepsis (29.8%). The mean ± SD values of age, dose, and the duration of TM-α administration were 64.7 ± 20.3 years, 297.3 ± 111.4 U/kg/day, and 5.6 ± 3.4 days, respectively. A total of 1,320 subjects (73.9%) received combined administration with other anticoagulants. Both coagulation and inflammation markers, such as fibrin/fibrinogen degradation products, prothrombin time ratio, thrombin-antithrombin complex, and C-reactive protein, as well as JAAM DIC, SIRS, and SOFA scores, significantly and simultaneously decreased after TM-α administration (*p* < 0.001). DIC resolution and 28-day survival rates were 44.4% and 66.0%, respectively. The 28-day survival rate decreased significantly according to the duration of DIC before TM-α administration (*p* < 0.001). Total adverse drug reactions (ADRs), bleeding ADRs, and serious bleeding adverse events occurred in 126 (7.1%), 98 (5.5%), and 121 (6.8%) subjects, respectively. On day 28, after the last TM-α administration available for an antibody test, only one patient was positive for anti-TM-α antibodies (0.11%).

**Conclusion:**

Our results suggest that TM-α is most effective for treating patients with sepsis-induced DIC when administered within the first 3 days after diagnosis.

## Background

In severe sepsis, disseminated intravascular coagulation (DIC) occurs in about 35% of patients [1]. The mortality rate is higher in patients with DIC (40% to 46.2%) than in patients without DIC (22.2% to 26.5%) [2, 3]; thus, the prognosis of sepsis is considered to be affected by the presence of DIC.

Thrombomodulin (TM) is a thrombin receptor on the endothelial cell surface that plays an important role in the regulation of intravascular coagulation [4]. Thrombin binds to TM, and the thrombin-TM complex then activates protein C to form activated protein C (APC), which cleaves and inactivates factors Va and VIIIa in the presence of protein S. Therefore, TM acts as a negative feedback regulator according to the amount of excessively generated thrombin. In addition to its anticoagulant properties, TM has an anti-inflammatory effect through the generation of APC, and more, directly regulates high mobility group box-1 (HMGB-1) [5] and endotoxin [6] through the lectin-like domain, independent of protein C activation.

Thrombomodulin alfa (TM-α; Recomodulin® Injection, Asahi Kasei Pharma Corporation, Tokyo, Japan) is a recombinant, human, soluble TM possessing an extracellular domain that includes a functional domain. The effects of TM-α on DIC have been examined in a multicenter, randomized, clinical trial in Japan [7, 8]. In patients with DIC associated with infection or hematological malignancy, resolution rates for DIC and bleeding symptoms were significantly better in the TM-α group than in the heparin group. TM-α was approved for use as a curative medicine for DIC for the first time in Japan in 2008. Since the launch of this product, a clinical use survey was conducted by continuous registration of all cases using TM-α up to specific target numbers (infection and hematological malignancy, ≥ 1,000 cases each; total, ≥ 3,000 cases). We have already reported the result of the all-case post-marketing surveillance (PMS) study of TM-α [9].

The present PMS sub-population analysis evaluated the effects and safety of TM-α in 1,787 sepsis-induced DIC patients who were prescribed TM-α according to the Japanese Association for Acute Medicine (JAAM) DIC criteria for early diagnosis of DIC.

## Methods

### Study design and patient population

**Figure 1:**
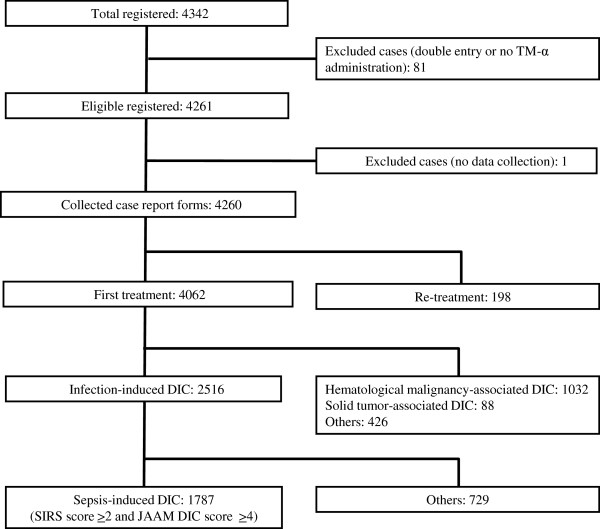
**Subject disposition.** *DIC* disseminated intravascular coagulation, *JAAM* Japanese Association for Acute Medicine, *SIRS* systemic inflammatory response syndrome, *TM-α* thrombomodulin alfa.

The subjects in the present observational study were a subset of those in the PMS reported by Mimuro et al. [9]. In response to requests for methods to improve the early diagnosis of DIC accompanying infections in the fields of emergency and ICU care, the JAAM DIC criteria aimed at achieving early diagnosis were announced in 2006 by the DIC Special Committees [10]. A total of 1,787 sepsis-induced DIC subjects were analyzed (Figure 1). From May 2008 to April 2010, 1,787 patients were registered in this study from 364 institutes, and DIC was diagnosed based on a score of 4 points on the JAAM DIC scoring system (see Appendix) [10]. The exclusion criteria were as follows: patients without SIRS (SIRS score ≤ 1), missing data for complete analysis, those with an unknown outcome, intracranial, respiratory, or continuous gastrointestinal bleeding, hypersensitivity to this agent, and pregnancy.

The data of patients re-treated with TM-α were excluded from this analysis. TM-α was administered by intravenous infusion for 30 min once a day at a dose of 380-U/kg body weight [7]. Since TM-α is excreted by the kidney, the dose of TM-α administered to subjects with renal dysfunction was reduced to 130-U/kg body weight. TM-α was infused after DIC was diagnosed, but the start of TM-α administration was not defined; therefore, each physician was free to determine the initial timing of TM-α infusion. In this PMS study, no limitation was placed on the period of TM-α administration and each physician individually judged the completion of TM-α administration. There was no limitation on the use of other anticoagulants, including antithrombin substitution, synthetic protease inhibitors (gabexate mesylate or nafamostat mesylate), heparin derivatives (heparin, dalteparin, or danaparoid sodium), or blood preparations such as fresh frozen plasma (FFP) and platelet concentrate, before and/or after TM-α administration.

Serum samples collected at baseline and on day 28 after the last TM-α administration were tested by enzyme-linked immunosorbent assay (ELISA) for anti-TM-α antibodies. This PMS study was conducted in accordance with the guidelines for Good Post-Marketing Surveillance Practices as required by the Japanese Ministry of Health, Labor, and Welfare. All subjects were treated according to the attending physicians' decisions, and no limitations were placed on the concomitant use of other anticoagulants or medicine for the treatment of underlying diseases and complications. In addition, personal data anonymization was carried out upon data collection. Therefore, approval of this surveillance by ethical committees and institutional review boards or informed consent acquisition was not necessary.

### Data collection

Organ dysfunction was assessed using the SOFA score. SIRS, sepsis, and septic shock were defined according to the American College of Chest Physicians/Society of Critical Care Medicine consensus conference [11] and Surviving Sepsis Campaign Guidelines 2008 [12], respectively. DIC resolution was defined as a total JAAM DIC score ≤ 3 by the day after the last TM-α administration. The JAAM DIC resolution rate was calculated using data from 1,152 subjects who were categorized as either ‘resolution’ or ‘non-resolution’. The survival rate was calculated based on the number of subjects who were alive 28 days after the initial TM-α administration (*n* = 1,771).

Subjects were followed until day 28 after the last administration. At the start of TM-α administration, information about the following 14 baseline characteristics was collected: age, sex, severity of underlying disease, history of bleeding, duration of DIC, kidney injury, liver injury, bleeding or symptoms of organ dysfunction, platelet count, fibrin/fibrinogen degradation products (FDP) or fibrinogen or antithrombin levels, and PT ratio. Furthermore, the JAAM DIC, SIRS, and SOFA scores were added as a factor relevant to sepsis [10]. Multiple logistic-regression analysis was then conducted using a total of 15 baseline characteristics excluding ‘severity of underlying disease’ and ‘liver injury’ , which are subjective observations.

Blood collection for clinical laboratory tests and coagulation studies was performed at the following three time points: before, during, and after TM-α administration. However, the TM-α administration period was excluded from the data analysis because the time varied by case. Safety data were coded using the preferred terms from version 13.1 of the Japanese version of the Medical Dictionary for Regulatory Activities (MedDRA/J) [13]. Definitions of adverse events (AEs) and adverse drug reactions (ADRs) were based on the International Conference on Harmonization of Technical Requirements for Registration of Pharmaceuticals for Human Use (ICH) guidelines [14]. The safety evaluation included serious bleeding AEs and all ADRs observed until 28 days after the last TM-α administration.

### Statistical analysis

In the descriptive analysis of baseline characteristics, numerical data are expressed as means ± standard deviation (SD) or medians (Q1–Q3; interquartile range). Statistical analysis was performed to compare values using the chi-square test, the Wilcoxon signed rank test, and the Cochran-Armitage test. To identify risk factors, DIC resolution and survival at 28 days after starting TM-α administration were stratified and tabulated by the 15 baseline characteristics. Differences in frequency by strata were tested for significance by the chi-square test. Relative risk factors for the DIC resolution rate and the survival rate after TM-α administration for DIC treatment were analyzed by multiple logistic regression analysis. Relative risk factors for serious bleeding AEs were identified using multiple logistic regression analysis. Odds ratios (ORs) with 95% confidence intervals (CIs) for selected variables were calculated. A value of *p* < 0.05 was considered significant. Statistical testing was performed with JMP version 9.0 software (SAS Institute, Cary, NC, USA).

## Results

### Subject demographics

A total of 1,787 patients were enrolled. Subject demographic characteristics are shown in Table 1. The mean ± SD age was 64.7 ± 20.3 years, and the median age was 70 (59–78, Q1-Q3) years. A total of 647 subjects (36.3%) were over 75 years old. The most frequent underlying disease at the time when TM-α was started was infectious focus-unknown sepsis (*n* = 533, 29.8%). The DIC score (median, Q1–Q3) was 6.0 (5.0–7.0). There were 816 (58.3%) subjects with a DIC score over 6 points and thus considered to be critically ill [15]. The SOFA and SIRS scores (median, Q1–Q3) were 11 (8.0–14.0) and 3.0 (3.0–4.0), respectively. Using the SOFA score, 553 subjects (50.8%) were severe cases, scoring over 11 points [16]. In regards to the timing of TM-α administration, 63.5% of subjects were treated with TM-α on the same day that they were diagnosed as having DIC, but 25.5% and 11.1% were started 1 to 2 days and over 3 days after being diagnosed as having DIC, respectively. The mean ± SD duration of TM-α administration was 5.6 ± 3.4 days, and the most frequent duration was 6 days (*n* = 589). The mean ± SD dose was 297.3 ± 111.4 U/kg/day, whereas the most common doses were near 380 (*n* = 1,008, 56.5%) and 130 (*n* = 424, 23.8%) U/kg/day.

**Table 1:** **Subject baseline demographics and characteristics**

Item		Subjects (***n***)	Rate (%)
Sex	Male	1038	58.1
	Female	749	41.9
Age (years)	0–14	86	4.8
	15–64	559	31.3
	65–74	491	27.5
	75–84	498	27.9
	≥ 85	149	8.3
	Unknown	4	0.2
Source of sepsis	Lungs	346	19.4
	Abdomen	396	22.2
	Urine	180	10.1
	Skin	48	2.7
	Focus-unknown	533	29.8
	Others	284	15.9
DIC score (JAAM criteria)	4	216	15.4
	5	368	26.3
	6	325	23.2
	7	191	13.6
	8	300	21.4
SOFA score	0–7	266	24.4
	8–10	270	24.8
	11–13	275	25.3
	≥ 14	278	25.5
SIRS score	2	394	24.4
	3	708	43.8
	4	513	31.8
Other anticoagulant treatment for DIC (Overall/after TM-α administration)		1,320/1,020	73.9/57.1
AT concentrate		876/476	49.0/26.6
Synthetic protease inhibitors		816/333	45.7/18.6
Heparin derivatives		427/212	23.9/11.9
Duration (days) of DIC before TM-α administration	0	1,134	63.5
	1	345	19.3
	2	110	6.2
	3	59	3.3
	4–6	78	4.4
	≥ 7	61	3.4

*AT* antithrombin, *DIC* disseminated intravascular coagulation, *JAAM* Japanese Association for Acute Medicine, *SIRS* systemic inflammatory response syndrome, *SOFA* Sequential Organ Failure Assessment, *TM-α* thrombomodulin alfa.

### Changes in coagulation and inflammation markers

**Figure 2:**
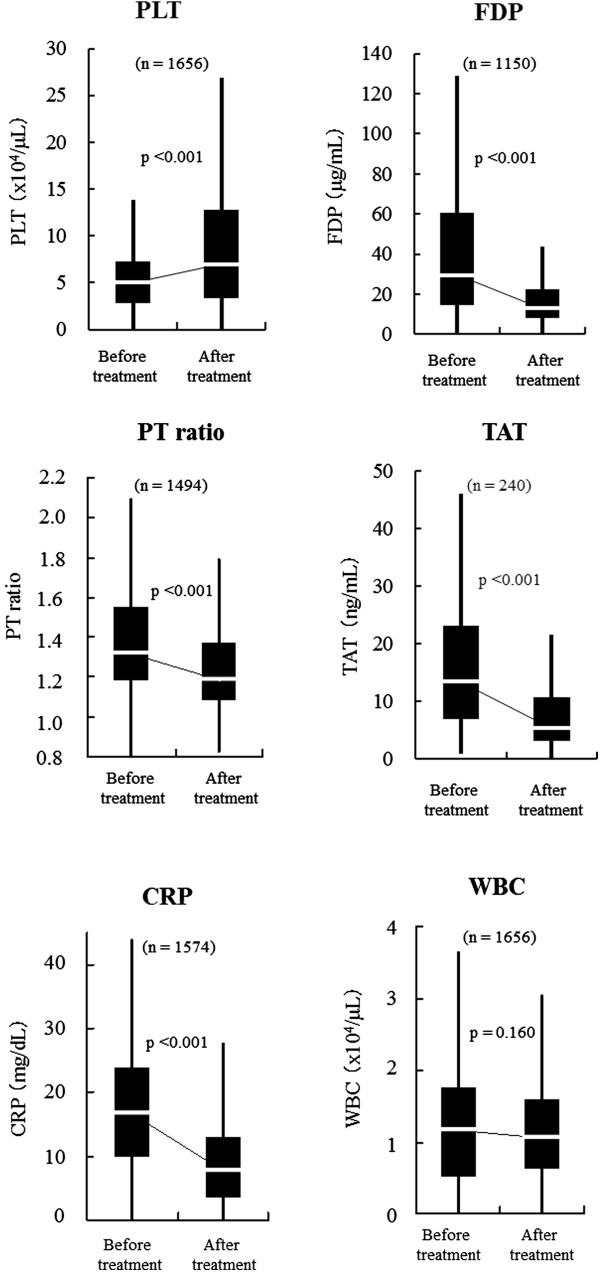
**Changes of coagulation and inflammation markers before and after TM-α administration.** *p* values: Wilcoxon signed-rank test. *CRP* C-reactive protein, *FDP* fibrin/fibrinogen degradation products, *PLT* platelet, *PT* prothrombin time, *TAT* thrombin-antithrombin, *TM-α* thrombomodulin alfa, *WBC* white blood cell.

Figure 2 shows the coagulation and inflammation markers before and after TM-α administration. All values are shown as median (Q1–Q3). The median platelet count increased significantly from 5.0 (2.8–7.3) to 7.0 (3.4–12.8) × 10^4^/μL (*p* < 0.001), and plasma levels of FDP (μg/mL), PT ratio, and TAT (ng/mL) decreased significantly from 29.5 (14.6–60.5) to 13.0 (8.0–22.3) (*p* < 0.001), 1.3 (1.2–1.6) to 1.2 (1.1–1.4) (*p* < 0.001), and 13.4 (7.0–23.1) to 5.3 (3.2–10.6) (*p* < 0.001), respectively. In the 1,076 subjects who had not been given platelet concentrates and FFP, the median platelet count increased significantly from 5.6 (3.3–7.7) to 8.5 (4.2–14.5) × 10^4^/μL (*p* < 0.001), and in 966 subjects, the PT ratio decreased from 1.3 (1.2–1.5) to 1.2 (1.1–1.3) (*p* < 0.001). CRP (mg/dL) levels decreased significantly from 16.7 (9.8–24.0) to 7.7 (3.7–13.2) (*p* < 0.001), according to the improvement of these coagulation parameters. White blood cell (WBC) counts did not change, even when the subjects who had both WBC and CRP levels measured (*n* = 1,574) were examined. Fibrinogen (mg/dL) levels decreased slightly, but significantly, from 370 (247–504) to 347 (239–472) (*p* < 0.001) before and after TM-α administration.

### Changes in the DIC, SIRS, and SOFA scores

**Figure 3:**
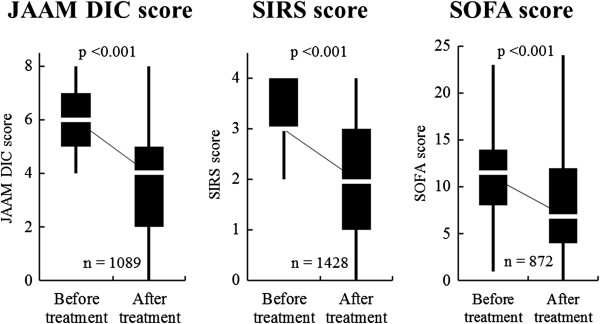
**Changes in JAAM DIC, SIRS, and SOFA scores before and after TM-α administration.** *p* values: Wilcoxon signed-rank test. *DIC* disseminated intravascular coagulation, *JAAM* Japanese Association for Acute Medicine, *SIRS* systemic inflammatory response syndrome, *SOFA* Sequential Organ Failure Assessment.

Changes in the DIC, SIRS, and SOFA scores before and after TM-α administration are shown in Figure 3. The DIC, SIRS, and SOFA scores decreased significantly from 6.0 (5.0–7.0) to 4.0 (2.0–5.0) (*p* < 0.001), 3.0 (3.0–4.0) to 2.0 (1.0–3.0) (*p* < 0.001), and 11.0 (8.0–14.0) to 7.0 (4.0–12.0) (*p* < 0.001), respectively. These findings indicate that recovery of the coagulation disorders and attenuation of both inflammation and organ dysfunction occurred simultaneously during TM-α administration.

### DIC resolution rate

Of the 1,152 subjects, DIC resolved in 512 after TM-α administration; therefore, the DIC resolution rate was 44.4%. The resolution rates decreased significantly in proportion to the severity of the DIC and SOFA scores before TM-α administration (*p* < 0.001) (Table 2). Comparing the subjects who were injected with 130 and 380 U/kg of TM-α, the resolution rates were 29.8% (78/262) and 51.7% (341/660), respectively; this difference was significant (*p* < 0.001). Other anticoagulants before TM-α administration did not affect the resolution rate (present 43.5% (225/517) vs. absent 45.2% (287/635), *p* = 0.724). A multiple logistic regression analysis showed that fibrinogen, JAAM DIC, and SOFA scores before TM-α administration were significant independent factors affecting the DIC resolution rate (Table 3).

**Table 2:** **The JAAM DIC resolution rate of DIC and SOFA scores before TM-α administration**

Factor		Total subjects (***n***)	DIC resolution		***p*** value^*^
Item	Score		Subjects (***n***)	Rate (%)	
JAAM DIC score before TM-α administration	Total	1,089	488	44.8	-
	4	164	93	56.7	< 0.001
	5	285	146	51.2	
	6	259	110	42.5	
	7	153	60	39.2	
	8	228	79	34.6	
SOFA score before TM-α administration	Total	768	348	45.3	-
	0–7	173	104	60.1	< 0.001
	8–10	192	94	49.0	
	11–13	209	91	43.5	
	≥ 14	194	59	30.4	

*Cochran-Armitage test.

**Table 3:** **Multiple logistic regression analysis of risk factors affecting the JAAM DIC resolution rate**

Item	Odds ratio (95% CI)		***p*** value
Fibrinogen before TM-α administration	1.002	(1.001–1.003)	< 0.0001
JAAM DIC score before TM-α administration	0.836	(0.743–0.939)	0.0026
SOFA score before TM-α administration	0.894	(0.857–0.932)	< 0.0001

A total of 693 DIC patients diagnosed by JAAM DIC criteria were analyzed by multiple logistic regression analysis; *CI* confidence interval, *DIC* disseminated intravascular coagulation, *JAAM* Japanese Association for Acute Medicine, *SOFA* Sequential Organ Failure Assessment, *TM-α* thrombomodulin alfa.

### Survival rate

**Figure 4:**
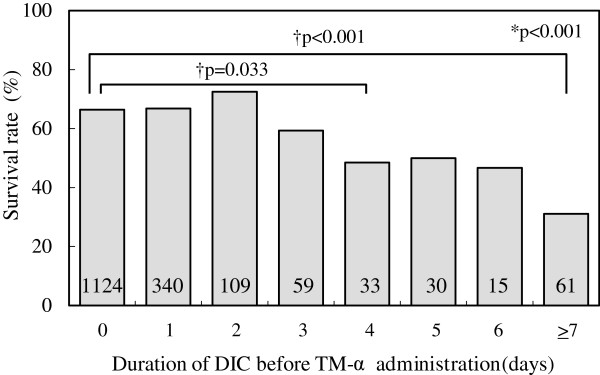
**Relationship between duration of DIC (days) before TM-α administration and survival rates.** The numbers in the columns represent the number of patients, excluding those with no data for survival. *Cochran-Armitage test, ^†^chi-square test. *DIC* disseminated intravascular coagulation, *TM-α* thrombomodulin alfa.

With respect to overall mortality, 1,144 of the 1,771 subjects survived 28 days after TM-α administration; therefore, the overall mortality rate was 35.4%. The survival rate decreased significantly in proportion to the duration of DIC before TM-α administration (*p* < 0.001) (Figure 4). More precisely, the 28-day survival rate was 66.4% when TM-α was injected on the same day that DIC was diagnosed, whereas it was 48.5% when TM-α was started 4 days later, and 31.1% when it was started 7 or more days after DIC was diagnosed. These differences were significant (*p* = 0.033 and *p* < 0.001, respectively). The survival rate decreased significantly in proportion to the severity of the SOFA score before TM-α, but not the DIC score (Table 4). The 28-day survival rate was 88.4% (449/508) in subjects whose DIC diagnosed by the JAAM criteria resolved after administration of TM-α, whereas it was 54.1% (343/643) in the subjects whose DIC continued. Therefore, resolution of DIC diagnosed by the JAAM criteria after TM-α administration strongly reflects the 28-day survival rate. Use of other anticoagulants before and after TM-α administration did not affect the survival rate (present vs. absent: 63.8% (485/760) vs. 65.2% (659/1011), *p* = 0.782, and 62.5% (288/461) vs. 65.3% (856/1310), *p* = 0.606, respectively). Survival rates in the subjects treated with 130 and 380 U/kg of TM-α were 58.5% (245/419) and 68.8% (688/1,000), respectively, with a tendency for improvement with 380 U/kg (*p* = 0.085). A multiple logistic regression analysis revealed that sex, duration of DIC, fibrinogen, and SOFA score before TM-α administration were significant independent factors related to mortality (Table 5).

**Table 4:** **The 28-day survival rate after TM-α administration of DIC and SOFA scores before TM-α administration**

Factor		Total subjects (***n***)	Survival rate		
Item	Score		Subjects (***n***)	Rate (%)	***p*** value^*^
JAAM DIC score before TM-α administration	Total	1,387	915	66.0	-
	4	215	147	68.4	0.630
	5	363	248	68.3	
	6	323	208	64.4	
	7	191	121	63.4	
	8	295	191	64.7	
SOFA score before TM-α administration	Total	1,077	712	66.1	-
	0–7	263	214	81.4	< 0.001
	8–10	266	192	72.2	
	11–13	270	174	64.4	
	≥14	278	132	47.5	

*Chi-square test.

**Table 5:** **Multiple logistic regression analysis of risk factors affecting the survival rate**

Item	Odds ratio (95% CI)		***p*** value
Sex (female/male)	1.546	(1.156–2.075)	0.0033
Duration of DIC before TM-α administration	0.931	(0.876–0.982)	0.0072
Fibrinogen before TM-α administration	1.001	(1.001–1.002)	0.0002
SOFA score before TM-α administration	0.874	(0.842–0.906)	< 0.0001

A total of 983 DIC patients diagnosed by JAAM DIC criteria were analyzed by multiple logistic regression analysis; *CI* confidence interval, *DIC* disseminated intravascular coagulation, *JAAM* Japanese Association for Acute Medicine, *SOFA* Sequential Organ Failure Assessment, *TM-α* thrombomodulin alfa.

### Safety

ADRs were observed in 126 subjects (7.1%), and bleeding ADRs and serious bleeding AEs occurred in 98 (5.5%) and 121 (6.8%) subjects, respectively (Table 6). The frequencies of bleeding ADRs and serious bleeding AEs in subjects treated with 130 and 380 U/kg were 5.9% and 4.9%, and 9.4% and 5.2%, respectively. Therefore, it is possible that the occurrence of these ADRs and AEs did not depend on the TM-α dose; additional research is needed to clarify this point. Of the 1,787 subjects, 121 had serious bleeding AEs, with gastrointestinal bleeding being the most frequently observed serious bleeding AE (2.7%, *n* = 48), followed by respiratory (1.0%, *n* = 18) and intracranial (0.8%, *n* = 15) bleeding, wound bleeding (0.6%, *n* = 11), and injection site bleeding (0.5%, *n* = 9).

**Table 6:** **ADR and AE incidences**

	Cases/Total	Incidence (%)
ADRs		
Total	126/1,787	7.1
Bleeding	98/1,787	5.5
AEs		
Serious bleeding	121/1,787	6.8

*ADRs* adverse drug reactions associated with administration of TM-α, *AEs* adverse events. ADRs and AEs were analyzed according to the Medical Dictionary for Regulatory Activities (MedDRA/J).

To investigate the risk factors before TM-α administration for serious bleeding AEs, a multiple logistic regression analysis was performed (Table 7). History of bleeding risk and pre-existing bleeding symptoms were especially significant independent risk factors for serious bleeding AEs (*p* < 0.0001, respectively). Of the 1,787 subjects in this study, 285 (15.9%) and 266 (14.9%) had a history of bleeding risk and pre-existing bleeding symptoms before TM-α administration, respectively. Therefore, in the 1,282 subjects with neither a history of bleeding risk nor pre-existing bleeding symptoms, the frequencies of bleeding ADRs and serious bleeding AEs were 3.7% (*n* = 48) and 4.3% (*n* = 55), respectively. Although the incidence of bleeding is relatively small, it is not rare. Bleeding ADRs did not affect survival [present vs. absent: 62.2% (61/98) vs. 64.7% (1,083/1,673), *p* = 0.815], but serious bleeding AEs did [present vs. absent: 38.0% (46/121) vs. 66.5% (1,098/1,650), *p* = 0.001]. No subject was found to have antibodies that neutralized TM-α activity.

**Table 7:** **Multiple logistic regression analysis of risk factors affecting serious bleeding AEs**

Item	Odds ratio (95% CI)		***p*** value
Sex (female/male)	0.631	(0.403–0.970)	0.0355
History of bleeding risk, presence	2.899	(1.839–4.504)	< 0.0001
Pre-existing bleeding symptoms, presence	2.983	(1.885–4.655)	< 0.0001
Fibrinogen before TM-α administration	0.997	(0.996–0.999)	< 0.0001

A total of 1,602 DIC patients diagnosed by JAAM DIC criteria were analyzed by multiple logistic regression analysis; *AE* adverse event, *TM-α* thrombomodulin alfa.

## Discussion

In this PMS sub-population analysis, in which DIC was diagnosed according to the JAAM DIC criteria and TM-α was administered at 4 days or later, the 28-day survival rate was significantly decreased as compared with TM-α administration within 3 days of diagnosis. These findings suggest that early use of TM-α could be useful for treating sepsis-induced DIC.

The survival rate in this PMS study was somewhat lower than that of the phase III clinical trial [7]. In the phase III clinical trial, patients on dialysis or with renal dysfunction were excluded because of severely impaired drug excretion from the kidney. In the present PMS sub-population analysis, subjects with renal dysfunction had their dose reduced to 130 U/kg according to the approved prescribing information, because TM-α is predominantly excreted by the kidney. The DIC resolution and survival rates were significantly lower in subjects given 130 U/kg (29.8% and 58.5%, respectively) than in those given 380 U/kg (51.7% and 68.8%, respectively) TM-α. However, those given the lower dose had renal dysfunction and were, therefore, more severely ill than those given the higher dose. Indeed, the subjects' SOFA scores before TM-α administration were 12.4 ± 4.0 with the lower dose and 9.7 ± 3.9 with the higher dose. This may be the one reason why the subjects treated with the lower dose had a poor DIC resolution rate. As a result, it appears that as the DIC period extended, subjects treated with the lower dose had a higher mortality.

The interaction between inflammation and coagulation involves significant cross-talk between systems [17], and the anti-inflammatory and anticoagulant effects of TM-α have been reported by Ito et al. [18]. Yamakawa et al. [19] reported improvement of rapid CRP and FDP with TM-α as compared with a non-treated TM-α control group. Recently, TM-α improved the elevated circulating levels of HMGB-1 in patients with sepsis-induced DIC. The rate of change in HMGB-1 was 48.1% in the TM-α-treated group and 213% in the non-TM-α-treated group (*p* = 0.086) [20]. Taken together, the anti-inflammatory effect of TM-α may explain the attenuation of HMGB-1 followed by reduction of CRP. These mechanisms, in part, suggest that TM-α improves outcomes in patients with sepsis-induced DIC. Indeed, Yamakawa et al. reported that the 90-day survival rate was improved by TM-α administration in these DIC patients [21].

On the other hand, as for the present result, the WBC count in association with inflammation was not affected by TM-α administration. WBC counts in patients with severe infection sometimes show levels below the normal range, which then increase according to the attenuation of inflammation. These phenomena may, in part, explain the unchanged WBC levels before and after TM-α administration.

The rates of serious bleeding events and intracranial hemorrhage were somewhat high in the present PMS sub-population analysis. Khan et al. reported that both serious bleeding events and intracranial hemorrhage in patients receiving activated protein C were significantly higher in the retrospective observation studies than in the randomized controlled trial (RCT) [22]. A recent TM-α global phase IIB study of sepsis-induced DIC reported that there were no differences between TM-α and placebo groups in the prevalence of serious major bleeding events (5.1% in the TM-α group vs. 4.6% in the placebo group) [23]. In the present PMS sub-population analysis, bleeding ADRs did not affect survival, but serious bleeding AEs did. Serious bleeding may partially depend on the severity of DIC, because the fibrinogen level before TM-α administration affected bleeding as a serious AE on multiple logistic regression analysis.

In order to estimate the frequency of anti-TM-α antibody production, especially neutralizing antibody that neutralizes the activity of TM-α and/or endothelial cell thrombomodulin, anti-TM-α antibody was measured before, and on day 28 after the last TM-α administration by ELISA for anti-TM-α antibodies. In the 1,787 subjects, there were 945 serum samples taken on day 28 after the last TM-α administration available for an antibody test, and only one patient was positive for anti-TM-α antibodies (0.11%). However, the subject was not found to possess antibodies that neutralized TM-α activity in an *in vitro* study. Thus, it appears that treatment with TM-α in this subject did not decrease the effect of TM-α.

There were several limitations in the present PMS sub-population analysis. First, this PMS study was an examination of a single arm with no comparison arms. Second, this PMS study was performed under daily clinical practice conditions, with restrictions on neither the treatment of underlying diseases nor the usage of other anticoagulants. However, this investigation offers new information about the effect and safety profile of TM-α for sepsis-induced DIC in daily clinical practice, and it collected prospective data and analyzed sepsis-induced DIC diagnosed according to the JAAM DIC criteria, which was not fully elucidated by the phase III clinical study. The findings of a recent retrospective cohort study revealed that TM-α administration improved sepsis-induced DIC diagnosed according to the JAAM DIC criteria [24]. Despite these limitations, our findings may have important implications. Further study is needed to confirm these findings.

## Conclusions

The present findings suggest that TM-α is most effective for treating patients with sepsis-induced DIC when administered within the first 3 days after diagnosis. To verify the curative effect of TM-α on sepsis-induced DIC, a large-scale RCT is required.

## Appendix

**The JAAM DIC criteria are presented in Table **8**.**

**Table 8:** **JAAM DIC criteria**

		Score
Systemic inflammatory response syndrome criteria*		
	≥ 3	1
	0–2	0
Platelet count, × 10^4^/μL		
	<8 or > 50% decrease within 24 h	3
	≥ 8 and < 12; or > 30% decrease within 24 h	1
	≥ 12	0
Prothrombin time (value of subject/normal value)		
	≥ 1.2	1
	< 1.2	0
Fibrin/fibrinogen degradation products, μg/mL		
	≥ 25	3
	≥ 10 and < 25	1
	< 10	0
Diagnosis		
	≥ 4 points	DIC

Scoring system for disseminated intravascular coagulation (DIC) established by the Japanese Association for Acute Medicine. *Criteria for systemic inflammatory response syndrome: temperature > 38°C or < 36°C; heart rate > 90 beats/min; respiratory rate > 20 breaths/min or PaCO_2_ < 32 torr (< 4.3 kPa); white blood cell counts > 12,000 cells/μL, < 4,000 cells/μL, or 10% immature (band) forms.

## Abbreviations

AE: adverse event
ADR: adverse drug reaction
APC: activated protein C
AT: antithrombin
CI: confidence interval
CRP: C-reactive protein
DIC: disseminated intravascular coagulation
ELISA: enzyme-linked immunosorbent assay
FDP: fibrin/fibrinogen degradation products
FFP: fresh frozen plasma
HMGB-1: high mobility group box-1
ICU: intensive care unit
JAAM: Japanese Association for Acute Medicine
MedDRA/J: Medical Dictionary for Regulatory Activities
OR: odds ratio
PLT: platelet
PMS: post-marketing surveillance
PT: prothrombin time
RCT: randomized controlled trial
SD: standard deviation
SIRS: systemic inflammatory response syndrome
SOFA: Sequential Organ Failure Assessment
TAT: thrombin-antithrombin
TM: thrombomodulin
TM-α: thrombomodulin alfa
WBC: white blood cell.

## References

[1] Levi M (2007). Disseminated intravascular coagulation. Crit Care Med.

[2] Bernard GR, Vincent JL, Laterre PF, LaRosa SP, Dhainaut JF, Lopez-Rodriguez A, Steingrub JS, Garber GE, Helterbrand JD, Ely EW, Fisher CJ (2001). Recombinant human protein C Worldwide Evaluation in Severe Sepsis (PROWESS) study group: Efficacy and safety of recombinant human activated protein C for severe sepsis. N Engl J Med.

[3] Warren BL, Eid A, Singer P, Pillay SS, Carl P, Novak I, Chalupa P, Atherstone A, Pénzes I, Kübler A, Knaub S, Keinecke HO, Heinrichs H, Schindel F, Juers M, Bone RC, Opal SM (2001). KyberSept Trial Study Group: Caring for the critically ill patient. High-dose antithrombin III in severe sepsis: a randomized controlled trial. JAMA.

[4] Esmon CT (2005). The interactions between inflammation and coagulation. Br J Haematol.

[5] Ito T, Kawahara K, Okamoto K, Yamada S, Yasuda M, Imaizumi H, Nawa Y, Meng X, Shrestha B, Hashiguchi T, Maruyama I (2008). Proteolytic cleavage of high mobility group box 1 protein by thrombin-thrombomodulin complexes. Arterioscler Thromb Vasc Biol.

[6] Shi CS, Shi GY, Hsiao SM, Kao YC, Kuo KL, Ma CY, Kuo CH, Chang BI, Chang CF, Lin CH, Wong CH, Wu HL (2008). Lectin-like domain of thrombomodulin binds to its specific ligand Lewis Y antigen and neutralizes lipopolysaccharide-induced inflammatory response. Blood.

[7] Saito H, Maruyama I, Shimazaki S, Yamamoto Y, Aikawa N, Ohno R, Hirayama A, Matsuda T, Asakura H, Nakashima M, Aoki N (2007). Efficacy and safety of recombinant human soluble thrombomodulin (ART-123) in disseminated intravascular coagulation: results of a phase III, randomized, double-blind clinical trial. J Thromb Haemost.

[8] Aikawa N, Shimazaki S, Yamamoto Y, Saito H, Maruyama I, Ohno R, Hirayama A, Aoki Y, Aoki O (2011). Thrombomodulin α in the treatment of septic patients complicated by disseminated intravascular coagulation: subanalysis from the phase 3 trial. Shock.

[9] Mimuro J, Takahashi H, Kitajima I, Tsuji H, Eguchi Y, Matsushita T, Kuroda T, Sakata Y (2013). Impact of recombinant soluble thrombomodulin (thrombomodulin alfa) on disseminated intravascular coagulation. Thromb Res.

[10] Gando S, Iba T, Eguchi Y, Ohtomo Y, Okamoto K, Koseki K, Mayumi T, Murata A, Ikeda T, Ishikura H, Ueyama M, Ogura H, Kushimoto S, Saitoh D, Endo S (2006). Japanese Association for Acute Medicine Disseminated Intravascular Coagulation (JAAM DIC) Study Group, Shimazaki S: A multicenter, prospective validation of disseminated intravascular coagulation diagnostic criteria for critically ill patients: comparing current criteria. Crit Care Med.

[11] Members of the American College of Chest Physicians/Society of Critical Care Medicine Consensus Conference committee (1992). American College of Chest Physicians/Society of Critical Care Medicine Consensus Conference: definition for sepsis and organ failure and guidelines for the use innovative therapies in sepsis. Crit Care Med.

[12] Dellinger RP, Levy MM, Carlet JM, Bion J, Parker MM, Jaeschke R, Reinhart K, Angus DC, Brun-Buisson C, Beale R, Calandra T, Dhainaut JF, Gerlach H, Harvey M, Marini JJ, Marshall J, Ranieri M, Ramsay G, Sevransky J, Thompson BT, Townsend S, Vender JS (2008). Zimmerman JL, for the International Surviving Sepsis Campaign Guidelines Committee, Vincent JL: Surviving Sepsis Campaign: international guidelines for management of severe sepsis and septic shock: 2008. Crit Care Med.

[13] **Northrop Grumman Corporation. MedDRA and MSSO: Northrop Grumman Corporation website** http://www.meddra.org; April 8, 2011

[14] **ICH Steering Committee. ICH Harmonised Tripartite Guideline: Clinical safety data management: definitions and standards for expedited reporting**. http://www.pmda.go.jp/ich/e/e2a_95_3_20e.pdf; April 8, 2011

[15] Gando S, Saitoh D, Ogura H, Mayumi T, Koseki K, Ikeda T, Ishikura H, Iba T, Ueyama M, Eguchi Y, Otomo Y, Okamoto K, Kushimoto S, Endo S (2009). Japanese Association for Acute Medicine Disseminated Intravascular Coagulation (JAAM DIC) Study Group, Shimazaki S: Disseminated intravascular coagulation (DIC) diagnosed based on the Japanese Association for Acute Medicine criteria is a dependent continuum to overt DIC in patients with sepsis. Thromb Res.

[16] Ferreira FL, Bota DP, Bross A, Mélot C, Vincent JL (2001). Serial evaluation of the SOFA score to predict outcome in critically ill patients. JAMA.

[17] Mosnier LO, Zolkovic BV, Griffin JH (2007). The cytoprotective protein C pathway. Blood.

[18] Ito T, Maruyama I (2011). Thrombomodulin: protectorate God of the vasculature in thrombosis and inflammation. J Thromb Haemost.

[19] Yamakawa K, Fujimi S, Mohri T, Matsuda H, Nakamori Y, Hirose T, Tasaki O, Ogura H, Kuwagata Y, Hamasaki T, Shimazu T (2011). Treatment effects of recombinant human soluble thrombomodulin in patients with severe sepsis: a historical control study. Crit Care.

[20] Kudo D, Shinozawa Y, Yamanouchi S, Endo T, Sato T, Furukawa H, Nomura R, Kushimoto S (2012). Treatment effect of thrombomodulin-α on septic disseminated intravascular coagulation (DIC): a historical cohort study (in Japanese). J Jpn Soc Intensive Care Med.

[21] Yamakawa K, Ogura H, Fujimi S, Morikawa M, Ogawa Y, Mohri T, Nakamori Y, Inoue Y, Kuwagata Y, Tanaka H, Hamasaki T, Shimazu T (2013). Recombinant human soluble thrombomodulin in sepsis-induced disseminated intravascular coagulation: a multicenter propensity score analysis. Intensive Care Med.

[22] Khan A, Agarwal R, Aggarwal AN, Gupta D (2010). Prevalence of serious bleeding events and intracranial hemorrhage in patients receiving activated protein C: a systematic review and meta-analysis. Respir Care.

[23] Vincent JL, Ramesh MK, Ernest D, Larosa SP, Pachl J, Aikawa N, Hoste E, Levy H, Hirman J, Levi M, Daga M, Kutsogiannis DJ, Crowther M, Bernard GR, Devriendt J, Puigserver JV, Blanzaco DU, Esmon CT, Parrillo JE, Guzzi L, Henderson SJ, Pothirat C, Mehta P, Fareed J, Talwar D, Tsuruta K, Gorelick KJ, Osawa Y, Kaul I (2013). A randomized, double-blind, placebo-controlled, phase 2b study to evaluate the safety and efficacy of recombinant human soluble thrombomodulin, ART-123, in patients with sepsis and suspected disseminated intravascular coagulation. Crit Care Med.

[24] Kato T, Sakai T, Kato M, Hagihara M, Hasegawa T, Matsuura K, Nakagawa T (2013). Recombinant human soluble thrombomodulin administration improves sepsis-induced disseminated intravascular coagulation and mortality: a retrospective cohort study. Thromb J.

